# Effect of feeding rice gluten meal with and without enzymes on hematobiochemical profile of broiler chickens

**DOI:** 10.14202/vetworld.2020.2062-2069

**Published:** 2020-10-03

**Authors:** Om Prakash Dinani, Pramod Kumar Tyagi, Jagbir Singh Tyagi, Subrat Kumar Bhanja, J. J. Rokade

**Affiliations:** Avian Nutrition and Feed Technology Division, ICAR-Central Avian Research Institute, Bareilly, Uttar Pradesh, India

**Keywords:** broiler, enzyme, hematology, rice gluten meal, serology

## Abstract

**Background and Aim:**

Blood and serological parameters are indicators of the health status of the birds and influenced by the type of feed and their nutrient composition. Scanty researches are available in rice gluten meal (RGM) regarding its effect of feeding with and without enzymes on hematology and serum biochemistry. This study was conducted for *in vitro* and *in vivo* investigation regarding feeding RGM without or with different enzymes on hematology and serum biochemistry of broiler chickens. The *in vitro* study was done to determine chemical composition of RGM used in the biological trial.

**Materials and Methods:**

A biological experiment with 384 broiler chicks was conducted to evaluate the effect of feeding RGM as soybean replacement without or with different enzymes on carcass characteristics in broiler chicken for 42 days. Two levels of RGM were taken (15% and 17.5%). Protease, xylanase, and multienzymes supplementation under different treatments were done.

**Results:**

The RGM feeding and enzyme supplementation or their interaction revealed no significant (p>0.05) effects on the hematological parameters and serological parameters of broiler chickens except the significant (p<0.05) effect of enzyme supplementation on serum albumin and triglyceride values. The higher albumin values were observed in xylanase and protease supplemented birds and lower triglyceride values were observed in xylanase supplemented birds.

**Conclusion:**

Thus, it may be concluded that RGM feeding at 15% or 17.5% inclusion level with or without enzymes had no adverse effect on hematobiochemical profile of broiler chickens.

## Introduction

Poultry production in India has taken a quantum leap in the past four decades. At present, the total poultry population in India is 851.81 million numbers which are 16.8% increased from the 19^th^ livestock census [1]. The feed is the major component in the production of poultry, as it constitutes 65-75% of the total recurring cost. Soybean meal is the major proteinic ingredient used in poultry diet. The estimated requirement for soybean meal will be 11.9 million tons in 2025. However, the net deficiency of soybean meal in the country is about 2.5 MMT annually. Due to the scarcity of soybean at a reasonable price, there is a need to utilize locally available alternate protein ingredients [2]. However, only a narrow range of feed ingredients are used due to lack of reliable data on their nutritive quality, feeding value, and safe or effective level of inclusion.

Rice tops the list of total cereal production in the country. India is the second-largest producer of rice in the world after China, producing approximately 109.7 MT rice in 2016-2017 [3]. About 92% of total rice production is used for human food and about 8% is used for livestock and poultry feed in the form of rice bran, deoiled rice bran, rice polish, and broken rice. Nowadays, certain newer rice by-products are available in appreciable quantities and cheaper rate that can be utilized as protein sources from rice processing industries such as rice gluten meal (RGM).

Hematology dealing study of blood plays a leading role in the growth and nutritional physiology. The blood and serum metabolites provide useful information on nutritional status and clinical investigation of an individual; hence, the WHO recommended the use of blood parameters for medical and nutritional assessments [4]. Blood and serological parameters are indicators of the health status of the birds and influenced by the type of feed and their nutrient composition. Serum biochemical parameters are indicators of the physiological, nutritional, and pathological status of birds and can be correlated to identify the impact of nutritional factors and additives supplied in the diet. Type and level of crude fiber in feed, their amino acid composition, and the type of incriminating factors present in the feed play a pivotal role affecting blood and serum parameters [5].

Enzyme supplementations in poultry diets are nutritionally, economically, and environmentally justified. However, limited information is available on the appropriate enzyme or their combination that is specific for broiler diets based on corn-soya diet and soybean meal partially replaced with RGM. Strategic development of suitable non-starch polysaccharide (NSP) enzyme combination based on the composition of NSP in diet (substrate specific preparation) will enhance the nutritive value of diets [6]. The estimated crude fiber content of RGM was 7.4% in this experiment. Crude fiber is a rich source of NSP. Protease supplementation has been done to improve the protein digestibility of RGM. Xylanase supplementation has been done to degrade the NSP component of RGM. Multienzymes supplementation has been done to improve overall digestibility RGM. Thus, different enzymes have been used to find out the most suitable combination with RGM.

Very scanty researches were done in RGM regarding its effect of feeding with and without enzymes on hematology and serum biochemistry [7-9]. In view of the above, a study was conducted for *in vitro* and *in vivo* investigation regarding feeding RGM without or with different enzymes on hematology and serum biochemistry of broiler chickens. The *in vitro* study was done to determine chemical composition of RGM used in the biological trial.

## Materials and Methods

### Ethical approval

The research work was carried out at the Division of Avian Nutrition and Feed Technology, Indian Council of Agricultural Research-Central Avian Research Institute (ICAR-CARI), Izatnagar, India. The study was carried out as per the guidelines and approval of the Institute Animal Ethical Committee (IAEC) and Committee for the Purpose of Control and Supervision of Experiments on Animals (CPCSEA). The IAEC/CPCSEA number is 452/01/ab/CPCSEA.

### Study period and location

The research was carried out at the Division of Avian Nutrition and Feed Technology, ICAR-Central Avian Research Institute (CARI), Izatnagar, India in the year 2017 (from 8 May to 19 June) for the period of 6 weeks biological trial duration.

### Experimental design

The experiment was conducted as per 3×4 factorial completely randomized design (CRD). A total of 384 broiler chicks (CARIBRO Vishal) of the same hatch with uniform weight were used in the experiment. The birds were randomly divided into 48 replicates of eight birds each. There were 12 different treatments with four replicates for each treatment. Hence, each treatment was allocated 32 birds. Two levels of RGM were taken, the best inclusion level from earlier experiments as first level (15%) and then adding over and above the best level of 2.5% RGM to this level with enzymes. The experimental layout for feeding different levels of rDDGS with or without enzymes is presented in Table-1.

**Table 1:** Experimental layout for feeding different levels of RGM with or without enzymes.

Experimental design			3×4 factorial CRD		
	
Treatment	Rice gluten meal (%)	Number of replicates	Birds/replication	Total	Enzymes
T1	0.0	4	8	32	-
T2	0.0	4	8	32	Xylanase
T3	0.0	4	8	32	Protease
T4	0.0	4	8	32	Multienzymes
T5	15	4	8	32	-
T6	15	4	8	32	Xylanase
T7	15	4	8	32	Protease
T8	15	4	8	32	Multienzymes
T9	17.5	4	8	32	-
T10	17.5	4	8	32	Xylanase
T11	17.5	4	8	32	Protease
T12	17.5	4	8	32	Multienzymes

CRD=Completely randomized design, RGM=Rice gluten meal

### Procurement of feed ingredients

The required quantities of the feed ingredients and supplements for the formulation of experimental diets, including RGM enzyme supplements xylanase, protease, and multienzymes were procured from the feed storage and processing section of ICAR-CARI, Izatnagar.

### Procurement of experimental eggs

In the study, required eggs of CARIBRO-VISHAL (white variety) were obtained from the Experimental Broiler Farm, ICAR-CARI, Izatnagar, India, and incubated at Experimental Hatchery Unit of the institute. Day-old broiler chicks of the same hatch with uniform weight wing banded were used in the experiments.

### Housing and management

Experimental day-old chicks of broiler chickens were randomly divided into different groups as per the experimental plan. The birds were housed in specially designed battery brooder cages and reared under standard management conditions. Experimental diets were offered *ad libitum*, mash feed to all groups of broiler birds for an experimental period of 6 weeks. The weighted amount of respective diets was offered to birds daily with every attempt to minimize feed spillage/wastage. Fresh and wholesome water were always made available to the birds throughout the experimental period. All management practices, including feeding, watering, lighting, and vaccination practices were kept identical for all the birds under different dietary treatments.

### Basal diets and laboratory analysis

Analyzed chemical composition of dietary ingredients (%) is presented in Table-2. Corn-soya meal-based basal diets to meet standard [10] for broiler chickens were formulated as pre-starter (Table-3), starter (Table-4), and finisher (Table-5). The diets along with all the used ingredients, including and RGM chemical analysis were done as per standard procedure [11]. *Isonitrogenous* and *isocaloric* diets were used for all experiments. The three commercial enzyme preparations (protease, xylanase, and multienzymes) were analyzed for different enzyme activities as per the standard method [12].

**Table 2:** Analyzed chemical composition of dietary ingredients (%) on as such basis.

Ingredients	Moisture	DM	CP	EE	CF	TA	NFE	Ca	P	GE (kcal/kg)	*ME (kcal/kg)
Maize	8.6	91.3	9	3.9	1.8	1.4	83.8	0.03	0.29	4447	3350
SBM	9.1	90.9	44.5	0.9	6.2	3.1	45.2	0.32	0.68	4097	2400
DORB	10.1	91.8	14	1.6	15.9	5.8	62.6	0.3	1.54	3854	2000
RGM	7.6	92.3	49.9	5.7	7.4	3.3	33.5	0.84	0.98	4742	3031
Soybean oil	-	-	-	-	-	-	-	-	-	8900	8450
Limestone powder	1.4	98.6	-	-	-	-	-	33.89	-	-	-
Marble chips	1.3	98.7	-	-	-	-	-	33.84	-	-	-
DCP	7.2	92.7	-	-	-	-	-	22.92	16.04	-	-

* Calculated value. DM=Dry matter, CP=Crude protein, EE=Ether extract, CF=Crude fiber, TA=Total ash, NFE=Nitrogen Free Extract, Ca=Calcium, P=Phosphorus, GE=Gross energy, ME=Metabolizable energy, SBM=Soybean meal, DORB=Deoiled rice bran, RGM=Rice gluten meal, DCP= Dicalcium phosphate

**Table 3:** Ingredients and nutrient composition (%) of pre-starter diets with or without enzymes for different levels of RGM.

Ingredients	T1	T2	T3	T4	T5	T6	T7	T8	T9	T10	T11	T12
Maize	54.42	54.42	54.42	54.42	59.40	59.40	59.40	59.40	60.00	60.00	60.00	60.00
SBM	38.40	38.40	38.40	38.40	20.70	20.70	20.70	20.70	17.80	17.80	17.80	17.80
RGM	0.00	0.00	0.00	0.00	15.00	15.00	15.00	15.00	17.50	17.50	17.50	17.50
Oil	3.00	3.00	3.00	3.00	0.70	0.70	0.70	0.70	0.40	0.40	0.40	0.40
LSP	1.40	1.40	1.40	1.40	1.30	1.30	1.30	1.30	1.30	1.30	1.30	1.30
DCP	1.82	1.82	1.82	1.82	1.95	1.95	1.95	1.95	1.95	1.95	1.95	1.95
Lysine	0.00	0.00	0.00	0.00	0.12	0.12	0.12	0.12	0.18	0.18	0.18	0.18
Methionine	0.20	0.20	0.20	0.20	0.06	0.06	0.06	0.06	0.06	0.06	0.06	0.06
Constant*	0.765	0.765	0.765	0.765	0.765	0.765	0.765	0.765	0.765	0.765	0.765	0.765
Enzyme	−	+	+	+	−	+	+	+	−	+	+	+
Total	100.01	100.01	100.01	100.01	100.00	100.00	100.00	100.00	100.00	100.00	100.00	100.00
Nutrient composition												
CP	21.99	21.99	21.99	21.99	22.06	22.06	22.06	22.06	22.07	22.07	22.07	22.07
Lysine	1.19	1.19	1.19	1.19	1.20	1.20	1.20	1.20	1.21	1.21	1.21	1.21
Methionine	0.52	0.52	0.52	0.52	0.52	0.52	0.52	0.52	0.54	0.54	0.54	0.54
Threonine	0.81	0.81	0.81	0.81	0.83	0.83	0.83	0.83	0.82	0.82	0.82	0.82
Ca	1.03	1.03	1.03	1.03	1.01	1.01	1.01	1.01	1.01	1.01	1.01	1.01
P	0.45	0.45	0.45	0.45	0.45	0.45	0.45	0.45	0.44	0.44	0.44	0.44
ME (kcal/kg)**	2998	2998	2998	2998	3001	3001	3001	3001	3001	3001	3001	3001
Cost (Rs./kg)	28.52	29.03	29.13	28.93	24.68	25.19	25.29	25.09	24.31	24.82	24.92	24.72

In pre-starter diet,

* constant 0.765 includes salt 0.4%, trace mineral premix 0.1%, vitamin premix 0.15%, Vitamin B complex 0.015%, choline chloride 0.05%, and toxin binder 0.05%. Trace mineral premix supplied mg/kg diet: Mn, 55; I, 1; Fe, 75; Zn, 60; Cu, 10; Se, 0.15; and Cr, 0.2. The vitamin premix supplied per kg diet: Vitamin A, 5000 IU; Vitamin D3, 2400 IU; Vitamin E,15; and Vitamin K, 1 mg. Vitamin B complex supplied per kg diet: Vitamin B_1_, 5 mg; Vitamin B_2_, 6 mg; Vitamin B_6_, 5 mg; Vitamin B^12^, 15 mcg; nicotinic acid, 35 mg; pantothenic acid, 12 mg; biotin 0.15 mg; and folic acid 0.5 mg. Choline chloride supplied per kg diet: Choline, 1300 mg. (As per ICAR, 2013)

** Calculated value. SBM=Soybean meal, RGM=Rice gluten meal, LSP=Limestone powder, DCP=Di-calcium phosphate, CP=Crude protein, Ca=Calcium, P=Phosphorus, ME=Metabolizable energy

**Table 4:** Ingredients and nutrient composition (%) of starter diets with or without enzymes for different levels of RGM.

Ingredients	T1	T2	T3	T4	T5	T6	T7	T8	T9	T10	T11	T12
Maize	55.63	55.63	55.63	55.63	60.70	60.70	60.70	60.70	61.62	61.62	61.62	61.62
SBM	37.10	37.10	37.10	37.10	19.20	19.20	19.20	19.20	16.20	16.20	16.20	16.20
RGM	0.00	0.00	0.00	0.00	15.00	15.00	15.00	15.00	17.50	17.50	17.50	17.50
Oil	3.50	3.50	3.50	3.50	1.20	1.20	1.20	1.20	0.80	0.80	0.80	0.80
LSP	1.35	1.35	1.35	1.35	1.32	1.32	1.32	1.32	1.32	1.32	1.32	1.32
DCP	1.55	1.55	1.55	1.55	1.70	1.70	1.70	1.70	1.70	1.70	1.70	1.70
Lysine	0.00	0.00	0.00	0.00	0.06	0.06	0.06	0.06	0.06	0.06	0.06	0.06
Methionine	0.10	0.10	0.10	0.10	0.03	0.03	0.03	0.03	0.03	0.03	0.03	0.03
Constant*	0.765	0.765	0.765	0.765	0.765	0.765	0.765	0.765	0.765	0.765	0.765	0.765
Enzyme	-	+	+	+	-	+	+	+	-	+	+	+
Total	100.00	100.00	100.00	100.00	100.0	100.0	100.0	100.0	100.00	100.00	100.00	100.00
Nutrient composition											
CP	21.52	21.52	21.52	21.52	21.51	21.51	21.51	21.51	21.50	21.50	21.50	21.50
Lysine	1.38	1.38	1.38	1.38	1.09	1.09	1.09	1.09	1.04	1.04	1.04	1.04
Methionine	0.48	0.48	0.48	0.48	0.49	0.49	0.49	0.49	0.50	0.50	0.50	0.50
Threonine	0.80	0.80	0.80	0.80	0.79	0.79	0.79	0.79	0.81	0.81	0.81	0.81
Ca	0.95	0.95	0.95	0.95	0.95	0.95	0.95	0.95	0.95	0.95	0.95	0.95
P	0.41	0.41	0.41	0.41	0.40	0.40	0.40	0.40	0.40	0.40	0.40	0.40
ME (kcal/kg)**	3050	3050	3050	3050	3050	3050	3050	3050	3051	3051	3051	3051
Cost (Rs./ kg)	28.03	28.53	28.63	28.43	24.43	25.03	24.93	24.83	23.86	24.46	24.36	24.26

In starter diet,

* constant 0.765 includes salt 0.4%, trace mineral premix 0.1%, vitamin premix 0.15%, Vitamin B complex 0.015%, choline chloride 0.05%, and toxin binder 0.05%. Trace mineral premix supplied mg/kg diet: Mn, 55; I, 1; Fe, 60; Zn, 60; Cu, 10; Se, 0.15; and Cr, 0.2. The vitamin premix supplied per kg diet: Vitamin A, 5000 IU; Vitamin D_3_, 2400 IU; Vitamin E, 15; and Vitamin K, 1 mg. Vitamin B complex supplied per kg diet: Vitamin B_1_, 4 mg; Vitamin B_2_, 6 mg; Vitamin B_6_, 5 mg; Vitamin B_12_, 15 mcg; nicotinic acid, 35 mg; pantothenic acid, 10 mg; biotin 0.15 mg; and folic acid 0.5 mg. Choline chloride supplied per kg diet: Choline, 1200 mg. (As per ICAR, 2013)

** Calculated value. SBM=Soybean meal, RGM=Rice gluten meal, LSP=Limestone powder, DCP=Dicalcium phosphate, CP=Crude protein, Ca=Calcium, P=Phosphorus, ME=Metabolizable energy

**Table 5:** Ingredients and nutrient composition (%) of finisher diets with or without enzymes for different levels of RGM.

Ingredients	T1	T2	T3	T4	T5	T6	T7	T8	T9	T10	T11	T12
Maize	62.00	62.00	62.00	62.00	67.07	67.07	67.07	67.07	67.97	67.97	67.97	67.97
SBM	31.30	31.30	31.30	31.30	13.40	13.40	13.40	13.40	10.40	10.40	10.40	10.40
RGM	0.00	0.00	0.00	0.00	15.00	15.00	15.00	15.00	17.50	17.50	17.50	17.50
Oil	3.22	3.22	3.22	3.22	0.90	0.90	0.90	0.90	0.50	0.50	0.50	0.50
LSP	0.00	0.00	0.00	0.00	0.00	0.00	0.00	0.00	0.00	0.00	0.00	0.00
DCP	1.45	1.45	1.45	1.45	1.60	1.60	1.60	1.60	1.60	1.60	1.60	1.60
Lysine	0.00	0.00	0.00	0.00	0.12	0.12	0.12	0.12	0.12	0.12	0.12	0.12
Methionine	0.06	0.06	0.06	0.06	0.00	0.00	0.00	0.00	0.00	0.00	0.00	0.00
Marble chips	1.20	1.20	1.20	1.20	1.14	1.14	1.14	1.14	1.14	1.14	1.14	1.14
Constant*	0.765	0.765	0.765	0.765	0.765	0.765	0.765	0.765	0.765	0.765	0.765	0.765
Total	100.00	100.00	100.00	100.00	100.00	100.00	100.00	100.00	100.00	100.00	100.00	100.00
Nutrient composition											
CP	19.51	19.51	19.51	19.51	19.50	19.50	19.50	19.50	19.50	19.50	19.50	19.50
Lysine	1.20	1.20	1.20	1.20	0.98	0.98	0.98	0.98	0.92	0.92	0.92	0.92
Methionine	0.41	0.41	0.41	0.41	0.43	0.43	0.43	0.43	0.44	0.44	0.44	0.44
Threonine	0.86	0.86	0.86	0.86	0.81	0.81	0.81	0.81	0.80	0.80	0.80	0.80
Ca	0.86	0.86	0.86	0.86	0.85	0.85	0.85	0.85	0.85	0.85	0.85	0.85
P	0.38	0.38	0.38	0.38	0.38	0.38	0.38	0.38	0.37	0.37	0.37	0.37
ME**	3100.3	3100.3	3100.3	3100.3	3099.1	3099.1	3099.1	3099.1	3099.3	3099.3	3099.3	3099.3
Cost (Rs./kg)	2672	2672	2672	2672	2584	2584	2584	2584	2526	2526	2526	2526

In finisher diet,

* constant 0.77 includes salt 0.4%, trace mineral premix 0.1%, vitamin premix 0.15%, Vitamin B complex 0.015%, choline chloride 0.05%, and toxin binder 0.05%. Trace mineral premix supplied mg/kg diet: Mn, 50; I, 1; Fe, 50; Zn, 60; Cu, 8; Se, 0.15; and Cr, 0.2. The vitamin premix supplied per kg diet: Vitamin A, 5000 IU; Vitamin D_3_, 2400 IU; Vitamin E,15; and Vitamin K, 0.8 mg. Vitamin B complex supplied per kg diet: Vitamin B_1_, 4 mg; Vitamin B_2_, 6 mg; Vitamin B_6_, 5 mg; Vitamin B_12_, 15 mcg; nicotinic acid, 30 mg; pantothenic acid, 10 mg; biotin 0.15 mg; and folic acid 0.5 mg. Choline chloride supplied per kg diet: Choline, 900 mg. (As per ICAR, 2013)

** Calculated value. SBM=Soybean meal, RGM=Rice gluten meal, LSP=Limestone powder, DCP=Dicalcium phosphate, CP=Crude protein, Ca=Calcium, P=Phosphorus, ME=Metabolizable energy

### Hematological parameters

Whole blood (around 2 mL) collection was employed carefully from the jugular vein into sterile vials with 1% ethylenediaminetetraacetic acid as an anticoagulant for hematological analyses. Eight birds (four males and four females) were chosen randomly from each treatment at the end of the trial on the 42^nd^ day. Abacus junior vet 5 hematoanalyzer was used for the analysis of blood profile. The parameters studied were total leukocyte count (TLC), differential leukocyte count (DLC), hemoglobin (Hb) (%), packed cell volume (PCV), mean corpuscular volume (MCV), mean corpuscular hemoglobin (MCH), mean corpuscular hemoglobin concentration (MCHC), RBC distribution width (RDWc), platelet count, mean platelet volume (MPV), and platelet distribution width (PDWc).

### Serum biochemical parameters

#### Whole blood collection

Whole blood (around 2 mL) collection was employed carefully from the jugular vein into sterile vials without any anticoagulant for biochemical analysis. Eight birds (four males and four females) were chosen randomly from each treatment at the end of the trial. Blood samples were centrifuged briefly at 1000 rpm for 10 min and supernatant, the serum was decanted and deep frozen (−20°C) till analysis. Serum samples were analyzed using commercial standard diagnostic kits using the standard protocol. Estimation of serum glucose was done by glucose oxidase peroxidase endpoint assay [13], total protein by modified biuret endpoint assay [14], serum albumin by modified Bromocresol Green method [15], and total cholesterol [16] and serum triglyceride [17] by standard methods. Serum enzyme alkaline phosphatase (ALP) by a method of Kind and King [18], aspartate amino transferase (AST)/serum glutamic-oxaloacetic transaminase (SGOT) and alanine amino transferase/serum glutamic pyruvic transaminase (SGPT) by a method of Reitman and Frankel [19] using commercial diagnostic kits.

### Statistical analysis

Data subjected to test of significance as per CRD were analyzed for mean, standard errors, and analysis of variance by Snedecor and Cochran [20] using Statistical Package for the Social Sciences (SPSS) 16.0 version (UNICOM Systems, California, USA) and comparison of means was done using Tukey’s test [21].

## Results

The data pertaining to feeding different levels of RGM with or without enzymes on hematological parameters of the broiler chickens are presented in Table-6. Blood profile was studied in terms of total erythrocyte count, TLC, DLC, platelet count, Hb %, PCV, MCV, MCH, MCHC, MPV, heterophils and leukocyte ratio, RDWc, and PDWc. The results revealed that no significant (p>0.05) difference was observed in blood profile between control and other different dietary treatments by incorporating different levels of rDDGS (0, 12.5, and 15%), enzymes (X, P, and M), and due to their interaction. All the blood parameters were found within normal physiological range as measured by Abacus junior vet 5 hematoanalyzer (Diatron, USA).

**Table 6:** Effect of feeding different level of RGM with or without enzymes on hematological parameters.

Treatment	RGM %	Enzyme	TLC	Heterophils	Lymph	Mono	Heterophils %	Lymph %	Mono %	H:L	TRBC	Hb	PCV	MCV	MCH	MCHC	RDWc	PLT	PLT%	MPV	PDWc
T1	0	-	13.8	3.5	8.3	1.2	24.8	59.9	8.4	0.42	2.5	11.8	28.9	116	47.8	41.0	17.5	27.8	0.28	8.8	30.5
T2	0	X	13.9	3.2	8.6	1.2	23.0	61.9	9.0	0.37	2.6	11.1	29.9	122	42.8	35.3	18.3	27.5	0.28	8.8	31.3
T3	0	P	13.8	3.8	7.9	1.1	27.1	56.7	8.3	0.48	2.4	11.0	27.4	115	45.8	40.3	16.8	29.8	0.30	9.3	32.3
T4	0	M	13.7	3.3	8.1	1.2	24.3	58.6	8.6	0.42	2.5	11.4	29.8	109	46.5	42.5	17.5	26.8	0.27	8.8	30.3
T5	15	-	14.3	3.7	8.1	1.3	25.4	56.6	8.7	0.45	2.5	10.9	29.2	117	43.5	37.3	15.8	30.0	0.31	8.8	32.3
T6	15	X	13.7	3.5	8.2	1.1	25.6	59.8	8.3	0.43	2.4	10.9	27.6	114	44.8	39.5	16.8	26.3	0.27	8.3	30.0
T7	15	P	14.1	3.6	8.3	1.1	25.2	58.8	8.0	0.43	2.6	11.4	29.1	113	44.5	39.3	15.3	25.8	0.26	8.8	29.0
T8	15	M	14.2	3.7	8.1	1.1	25.2	57.1	8.0	0.44	2.5	11.5	31.3	125	45.8	36.8	16.8	26.8	0.27	8.5	30.0
T9	17.5	-	13.8	3.7	7.8	1.2	26.3	56.6	8.3	0.47	2.5	11.3	29.0	119	46.0	38.8	16.5	29.0	0.30	9.3	29.0
T10	17.5	X	13.2	3.5	7.5	1.1	26.6	56.7	8.5	0.47	2.4	10.8	28.2	117	44.8	38.3	16.0	23.3	0.24	8.8	29.3
T11	17.5	P	13.3	3.2	7.9	1.1	24.2	59.0	8.5	0.41	2.5	12.2	29.8	117	48.3	41.0	15.0	26.8	0.27	8.8	30.0
T12	17.5	M	13.6	3.5	7.9	1.2	25.5	57.8	8.9	0.44	2.4	10.7	29.8	124	44.8	36.3	16.8	29.0	0.30	8.5	29.0
Pooled SEM			0.16	0.06	0.11	0.02	0.33	0.41	0.14	0.01	0.03	0.15	0.33	1.16	0.64	0.64	0.28	0.53	0.01	0.08	0.34
RGM																					
0			13.8	3.4	8.2	1.2	24.8	59.3	8.6	0.42	2.5	11.3	29.0	115	45.7	39.8	17.5	27.9	0.28	8.9	31.1
15			14.1	3.6	8.2	1.2	25.3	58.1	8.2	0.44	2.5	11.2	29.3	117	44.6	38.2	16.1	27.2	0.28	8.6	30.3
17.5			13.5	3.5	7.8	1.1	25.6	57.5	8.6	0.45	2.5	11.2	29.2	119	45.9	38.6	16.1	27.0	0.28	8.8	29.3
Enzyme																					
-			14.0	3.6	8.1	1.2	25.5	57.7	8.5	0.45	2.5	11.3	29.0	117	45.8	39.0	16.6	28.9	0.29	8.9	30.6
X			13.6	3.4	8.1	1.2	25.0	59.5	8.6	0.42	2.5	10.9	28.6	118	44.1	37.7	17.0	25.7	0.26	8.6	30.2
P			13.8	3.5	8.0	1.1	25.5	58.2	8.3	0.44	2.5	11.5	28.8	115	46.2	40.2	15.7	27.4	0.28	8.9	30.4
M			13.8	3.5	8.0	1.2	25.0	57.8	8.5	0.44	2.5	11.2	30.3	119	45.7	38.5	17.0	27.5	0.28	8.6	29.8
Significance																		
RGM			NS	NS	NS	NS	NS	NS	NS	NS	NS	NS	NS	NS	NS	NS	NS	NS	NS	NS	NS
Enzyme			NS	NS	NS	NS	NS	NS	NS	NS	NS	NS	NS	NS	NS	NS	NS	NS	NS	NS	NS
Interaction			NS	NS	NS	NS	NS	NS	NS	NS	NS	NS	NS	NS	NS	NS	NS	NS	NS	NS	NS

Values bearing different superscripts within the column differ significantly *(p<0.01), **(p<0.05) and NS=Non-significant (p>0.05). RGM=Rice gluten meal, TLC=Total leukocyte count, TRBC=Total red blood count, Hb=Hemoglobin, PCV=Packed cell volume, MCV=Mean corpuscular volume, MCH=Mean corpuscular hemoglobin, MCHC=Mean corpuscular hemoglobin concentration, RDWc=Red blood count distribution width, PLT=Platelet, MPV=Mean platelet volume, PDWc=Platelet distribution width, SEM=Standard error of the mean, X=Xylanase, P=Protease, M=Multienzymes

The results pertaining to the influence of different levels of RGM feeding with or without enzymes to broilers on serological parameters are presented in Table-7. Effect of feeding different levels of RGM (0.15 and 17.5%) and their interaction with or without enzymes (X, P, and M) on serum glucose, total protein, albumin (A), globulin (G), A:G ratio, cholesterol, triglyceride, serum enzymes SGOT, SGPT, and ALP did not exhibit any significant (p>0.05) difference as compared to different dietary treatments and control. Effect of feeding without or with enzymes (xylanase, protease, and multienzymes) on serological parameters did not exhibit any significant (p>0.05) difference except serum albumen and triglyceride levels. Serum albumin was significantly (p<0.05) higher in protease and multienzymes supplemented groups as compared to without enzymes, but they (P and M) did not show any significant difference (p>0.05) from xylanase enzyme groups. Serum triglyceride was significantly (p<0.05) lower in xylanase enzyme supplemented groups as compared to protease and multienzymes groups, but xylanase groups did not show any significant difference (p>0.05) from without enzyme groups. However, serum triglyceride was significantly (p<0.05) higher in multienzyme groups as compared to xylanase and without enzyme groups, but multienzyme groups did not show any significant difference (p>0.05) from protease enzyme groups.

**Table 7:** Effect of feeding different level of RGM with or without enzymes on serological parameters.

Treatment	RGM %	Enzyme	Glucose	Protein	Albumin	Globulin	A:G	Cholesterol	Triglyceride	SGOT	SGPT	ALP
T1	0	-	204	4.5	1.80	2.73	0.67	119	104	182	12.7	62.2
T2	0	Xylanase	209	4.8	1.83	2.82	0.70	113	102	182	12.8	68.8
T3	0	Protease	208	4.8	1.93	3.00	0.60	118	98	184	12.5	67.8
T4	0	Multienzymes	210	4.8	1.85	2.97	0.62	114	94	186	12.7	63.7
T5	15	-	203	4.7	1.78	2.93	0.62	121	100	182	13.2	59.8
T6	15	Xylanase	209	4.7	1.92	2.90	0.63	119	99	182	12.3	61.2
T7	15	Protease	212	4.6	1.80	2.72	0.72	114	99	185	14.3	70.7
T8	15	Multienzymes	212	4.8	1.85	2.97	0.63	123	105	184	13.2	65.5
T9	17.5	-	205	4.4	1.77	2.67	0.68	124	102	182	13.3	60.8
T10	17.5	Xylanase	205	4.3	1.92	2.63	0.63	122	97	184	13.0	64.5
T11	17.5	Protease	212	4.9	1.63	2.97	0.65	123	105	182	14.3	72.0
T12	17.5	Multienzymes	208	4.5	1.73	2.75	0.63	113	100	186	12.2	60.8
		Pooled SEM	0.83	0.04	0.02	0.03	0.01	0.95	1.02	0.40	0.16	1.09
		RGM										
		0	207	4.7	1.86	2.83	0.66	116	102	183	12.7	66.1
		15	208	4.8	1.82	2.96	0.62	117	97	183	12.8	62.3
		17.5	211	4.7	1.86	2.85	0.66	120	101	184	13.4	66.6
		Enzyme										
		-	207	4.6	1.77^a^	2.85	0.63	119	96^a,b^	182	12.8	64.7
		X	210	4.8	1.87^b^	2.86	0.68	116	95^a^	185	12.6	63.1
		P	208	4.7	1.94^b^	2.83	0.68	116	102^b,c^	184	13.6	70.3
		Multienzymes	209	4.8	1.80^ab^	2.98	0.62	119	104^c^	183	12.8	61.8
		Significance									
		RGM	NS	NS	NS	NS	NS	NS	NS	NS	NS	NS
		Enzyme	NS	NS	p<0.05	NS	NS	NS	p<0.05	NS	NS	NS
		Interaction	NS	NS	NS	NS	NS	NS	NS	NS	NS	NS

Values bearing different superscripts within the column differ significantly *(p<0.01), **(p<0.05) and NS=Non-significant (p>0.05). RGM=Rice gluten meal, SGOT=Serum glutamic-oxaloacetic transaminase, SGPT=Serum glutamic pyruvic transaminase, ALP=Alkaline phosphatase, SEM=Standard error of the mean

## Discussion

Our results are in agreement with Wani *et al*. [9] in terms of hematological parameters who reported no significant (p>0.05) difference in Hb and PCV by incorporating 17.5% RGM with or without protease supplementation. No other references are available regarding the effect of feeding RGM on blood profile. Thus, it may be concluded that RGM up to 17.5% level with or without enzymes (X, P, and M) inclusion level did not show any adverse effect on hematological parameters. Initial research findings of Metwally and Farahat [7] showed that RGM can be included up to 12.5% level in broiler chickens and up to 20% level in broiler chickens without affecting growth performance as per Wani *et al*. [9].

Our results are in agreement with Metwally and Farhat [7], Kumar *et al*. [8], and Wani *et al*. [9]. Metwally and Farhat [7] reported no significant (p>0.05) difference in serum biochemical parameters (serum lipid profile, glucose, total protein, albumin, and globulin) up to the addition of 12.5% RGM in the diet of broiler chicken. Kumar *et al*. [8] also reported no significant (p>0.05) difference in the serological variables (glucose, blood urea nitrogen, plasma proteins, and non-esterified fatty acids) on addition of RGM up to 21% level in the diet of growing dairy calves. Wani *et al*. [9] reported no significant (p>0.05) difference in serological parameters by feeding different levels of RGM up to 20% level with or without protease enzyme supplementation.

Thus, RGM supplementation had not changed the hematological parameters with or without enzymes supplementation. An only serological parameter in terms of serum albumin and triglyceride values was affected in this study. No anti-nutritional factor is present in RGM since no negative effect was seen due to RGM inclusion. This might be due to protein quality and amino acids composition of RGM, type of crude fiber present in RGM, and type of enzyme supplementation. The higher albumin values were observed in xylanase and protease supplemented birds and lower triglyceride values were observed in xylanase supplemented birds. This might be due to better protein digestibility of RGM by protease supplementation since RGM is poor quality protein as compared to soybean meal. Xylanase enzyme supplementation breaks NSP present in the RGM as a crude fiber component. This NSP may be degraded due to xylanase supplementation which may be associated with a reduction in triglyceride value in RGM diets.

## Conclusion

Thus, it may be concluded that RGM feeding at 15% or 17.5% inclusion level with or without enzymes had no adverse effect on hematobiochemical profile of broiler chickens.

## Authors’ Contributions

PKT conceived the work. SKB designed the experiments. OPD collected samples and performed the experiments. JST performed the statistical analysis. JJR helped during experiments. All authors read and approved the final manuscript.

## References

[1] Livestock Census 20^th^ (2017). Department of Animal Husbandary, Dairying and Fisheries. Government of India, New Delhi.

[2] Mandal A.B (2017). Challenges of Feed Industries for Sustainable Poultry Production. IPSACON, Lead Paper and Souvenir, Bangalore.

[3] Agricultural Statistics (2018). Agricultural Statistics at a Glance, Department of Agriculture, Cooperation and Farmers Welfare. Government of India, New Delhi.

[4] WHO (1963). World Health Organization, Technical Report Series. No. 842 (Expert Committee on Medical Assessment and Nutritional Status). WHO, Geneva.

[5] Chessson A (2001). Non-starch polysaccharide degrading enzymes in poultry diets:Influence of ingredients on the selection of activities. World Poult. Sci.

[6] Lazaro R, Latorre M.A, Medel P, Gracia M, Mateos G.G (2004). Feeding regimen and enzyme supplementation to rye-based diets for broilers. Poult. Sci.

[7] Metwally A, Farahat M (2015). Nutritive value and feeding of rice gluten meal in broiler chickens. Res. Opin. Anim. Vet. Sci.

[8] Kumar R, Thakur S.S, Mahesh M.S (2016). Rice gluten meal as an alternative by-product feed for growing dairy calves. Trop. Anim. Health Prod.

[9] Wani M.A, Pramod K.T, Praveen K.T, Sheikh S.A, Dinani O.P, Hazarika R, Bhanja S.K, Mandal A.B (2017). Effect of rice gluten meal as protein source in the diet of broiler chicken:Immunity, gut microbial count, haematology and serum biochemical parameters. Ind. J. Poult. Sci.

[10] ICAR. (2013) Nutrient Requirements of Animals-poultry.

[11] AOAC (2000). Official Methods of Analysis. AOAC, Washington, DC.

[12] Kamra D.N, Agarwal N (2003). Techniques in Rumen Microbiology. IVRIDU, Izatnagar.

[13] Trinder P (1960). Enzymatic methods for glucose determination. Ann. Clin. Biochem 1969.

[14] Doumas B.T, Watson W.A, Biggs H.G (1971). Albumin standards and the measurement of serum albumin with bromocresol green. Clin. Chim. Acta.

[15] Gustafsson J.E (1978). Automated serum albumin determination by use of the immediate reaction with bromocresol green reagent. Clin. Chem.

[16] Wybenga D.R, Pileggi V.J (1970). Estimation of cholesterol. Clin. Chem.

[17] Fossati P, Lorenzo P (1982). Serum triglycerides determined calorimetrically with an enzyme that produces hydrogen peroxide. Clin. Chem.

[18] Kind P.R.N, King E.J (1954). Estimation of plasma phosphatase by determination of hydrolysed phenol with aminoantipyrine. J. Clin. Pathol.

[19] Reitman S, Frankel S.A (1957). Colorimetric test for determination of serum glutamic oxaloacetic and glutamic pyruvic transaminase. Am. J. Clin. Pathol.

[20] Snedecor G.W, Cochran W.G (2004). Statistical Methods.

[21] Tukey J (1949). Comparing individual means in the analysis of variance. Biometrics.

